# Jane: a new tool for the cophylogeny reconstruction problem

**DOI:** 10.1186/1748-7188-5-16

**Published:** 2010-02-03

**Authors:** Chris Conow, Daniel Fielder, Yaniv Ovadia, Ran Libeskind-Hadas

**Affiliations:** 1 Department of Computer Science, Harvey Mudd College, Claremont CA, USA; 2 Department of Computer Science, California State Polytechnic University, Pomona, CA, USA

## Abstract

**Background:**

This paper describes the theory and implementation of a new software tool, called *Jane*, for the study of historical associations. This problem arises in parasitology (associations of hosts and parasites), molecular systematics (associations of orderings and genes), and biogeography (associations of regions and orderings). The underlying problem is that of reconciling pairs of trees subject to biologically plausible events and costs associated with these events. Existing software tools for this problem have strengths and limitations, and the new *Jane *tool described here provides functionality that complements existing tools.

**Results:**

The *Jane *software tool uses a polynomial time dynamic programming algorithm in conjunction with a genetic algorithm to find very good, and often optimal, solutions even for relatively large pairs of trees. The tool allows the user to provide rich timing information on both the host and parasite trees. In addition the user can limit host switch distance and specify multiple host switch costs by specifying regions in the host tree and costs for host switches between pairs of regions. *Jane *also provides a graphical user interface that allows the user to interactively experiment with modifications to the solutions found by the program.

**Conclusions:**

*Jane *is shown to be a useful tool for cophylogenetic reconstruction. Its functionality complements existing tools and it is therefore likely to be of use to researchers in the areas of parasitology, molecular systematics, and biogeography.

## Background

One widely-used approach to the study of host-parasite relationships involves reconciling host and parasite phylogenetic trees via *event-cost methods*. In this approach, each event in the parasite phylogeny is mapped onto the host tree and a mapping is sought that minimizes the total cost with respect to a given cost metric. Such mappings allow us to examine sets of events that may have led to the coevolution of the host and parasite phylogenies and are the basis of statistical tests for assessing congruence.

**Figure 1:**
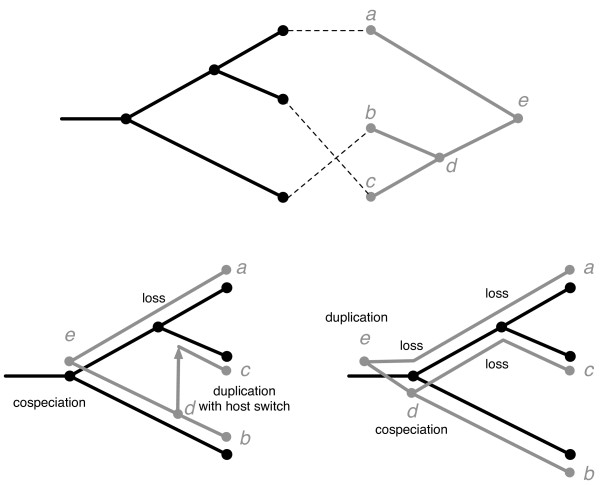
**Top: A simple tanglegram with host tree in black at left and parasite tree in gray on right**. The associations *φ *between tips is shown in dotted lines. Bottom: Two possible reconstructions that explain the relationship between *H *and *P *with events labeled.

Specifically, in the *cophylogeny reconstruction problem *we are given a host tree *H*; a parasite tree *P*; a function *φ *mapping the leaves or "tips" of *P*, representing the extant taxa, to the tips of *H*; and costs associated with each of four biologically plausible operations: cospeciation, duplication, host switching, and loss (Figure 1). Cospeciation occurs when a vertex (speciation event) in the parasite tree is associated with a vertex (speciation event) in the host tree. Duplication occurs when a vertex in the parasite tree is associated with an edge in the host tree. This event implies that the parasite lineage speciated independently of the host lineage. Host switching occurs when a duplication event is accompanied by one of the two descendants of the parasite vertex switching to an edge in a different part of the host tree. Once the parasite vertices are mapped onto the host tree, loss occurs when the path between a parasite vertex and its child passes through a host vertex. The objective is to find a least cost association of the trees that can be constructed with these four types of events.

We have recently shown that the cophylogeny reconstruction problem is NP-complete [1,2], and thus polynomial-time algorithms that find optimal solutions are unlikely to exist. Therefore, heuristics are required to find good, but not necessarily optimal, solutions.

A number of computational approaches for the cophylogeny reconstruction problem have been proposed and implemented in software. TreeFitter [3] and Component [4] were the first two programs for cophylogeny reconstruction. TreeFitter, according to its documentation, employs two methods: one provides a lower bound on the optimal solution but may introduce invalid solutions due to inconsistent host-switching events [3] while the other method is not described in detail but reportedly finds upper bounds on the cost. This software runs only on a limited number of platforms, some no longer available. While Component has several useful features, it does not consider host switching events and such events are known to be important in coevolution.

The computational intractability of the cophylogeny reconstruction problem is, in fact, due to host switching events. If host switching events are not considered then the problem can be solved optimally in polynomial time by a simple greedy algorithm.

**Figure 2:**
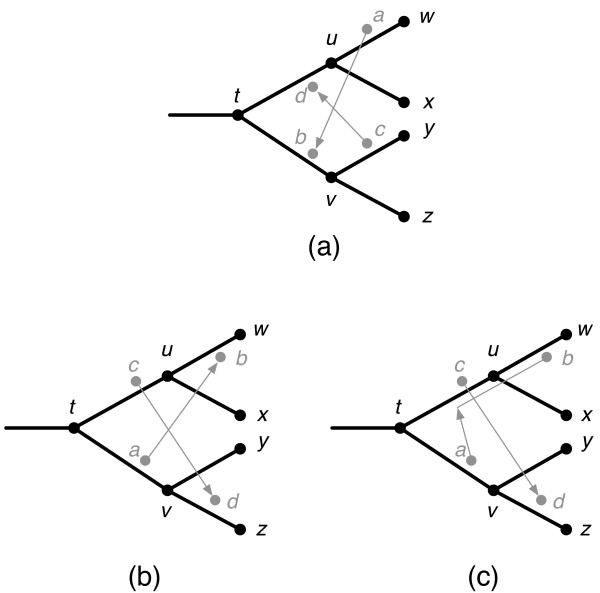
**(a) Strongly incompatible host switching events**. Parasite *a *on edge (*u*, *w*) switches to child *b *on edge (*t*, *v*) implying that *v *occurs after *u*. Similarly, parasite *c *on edge (*v*, *y*) switches to child *d *on edge (*t*, *u*) implying that *u *occurs before *v*. This results in an irreconcilable timing conflict. (b) Weakly incompatible host switching events. Parasite *a *on edge (*t*, *v*) switches to child *b *on edge (*u*, *w*) implying that *a *occurs after *u *and thus after *c*. Similarly, parasite *c *on edge (*t*, *u*) switches to child *d *on edge (*v*, *z*) implying that *c *occurs after *v *and thus after *a*. (c) This conflict can be resolved, for example, by moving one of the landing sites of a host switch earlier in time, incurring an additional loss event at *u*.

Host switching events can induce complex timing relationships between events. Figure 2(a) shows how a set of host switching events can result in a solution that is not valid because of an inconsistent sequence of timing events. Such a set of host switching events is called *strongly incompatible *[5]. In other cases, a set of host switching events may cause timing inconsistencies that can be resolved by moving the landing sites of one or more host switches to an earlier time at the expense of adding extra loss events. Such a set of switching events is called *weakly incompatible *[5]. Figure 2(b) shows a set of associations with weakly incompatible host switches and Figure 2(c) shows how these host switches can be modified to construct a valid mapping.

In seminal work on this problem, Charleston developed a data structure and algorithm called Jungles that solves the cophylogeny reconstruction problem optimally [5]. The Jungles approach discards all solutions with strong incompatibilities and optimally resolves weak incompatibilities. Jungles are implemented in the TreeMap software package [6]. While TreeMap is powerful and feature-rich, the worst-case time complexity of the Jungles algorithm is inherently exponential in the size of the host and parasite trees. Therefore, TreeMap is very useful for relatively small trees but it cannot be used for larger trees (e.g. pairs of trees with 25 tips each run for over two days on a commodity personal computer and eventually exceed available memory).

More recently, Merkle and Middendorf have proposed a heuristic for the cophylogeny reconstruction problem [7] and this heuristic has been implemented in the Tarzan software tool [8]. Tarzan is very fast (e.g. running in under one second on trees with 50 tips on a commodity personal computer) and in some cases can produce solutions that can be shown to be optimal. In particular, if Tarzan does not encounter any weak timing incompatibilities then, in theory, the solutions are optimal. Unfortunately, Tarzan occasionally reports solutions that are putatively optimal but are in fact incorrect due to weak or strong timing incompatibilities [9]. When Tarzan finds weak timing incompatibilities, it uses a heuristic to resolve them and thus cannot guarantee that the resulting solutions are optimal. In some cases, Tarzan encounters strong timing incompatibilities and reports that it cannot find a solution when solutions do exist.

In spite of the limitations described above, Tarzan has some important and unique features. In particular, Tarzan allows approximate times to be specified for divergence events in the host and parasite trees, thus restricting the solution space to mappings that are plausible for known timing of events. Specifically, nodes in the host and parasite trees can be partitioned into "time zones" and a node in the parasite tree can only be mapped to a region in the host tree in the same time zone. Since accurate timing is notoriously difficult to establish, Tarzan can allow a node to be associated with a range of time zones rather than a single time zone. However, due to the complexity of solving trees with time zones, Tarzan allows only parasite nodes to have time zone ranges. Even with this restriction, Merkle and Middendorf have demonstrated the value of time zone information by applying Tarzan to several host-parasite problems in the literature.

In this paper, we describe a new approach to the cophylogeny reconstruction problem and a software package called *Jane *that implements our technique. (The name "Jane" is used to indicate that this tools is complementary to Tarzan.) Specifically, Jane uses a dynamic programming algorithm [1] that finds optimal solutions in polynomial time for any fixed relative ordering, or "timing", of the vertices in the host tree. Since there are many possible timings of the events in the host tree, Jane applies a genetic algorithm that maintains a set (or "population" in the language of genetic algorithms) of timings of the host tree, uses the dynamic programming algorithm to solve the problem optimally for each timing in the set in order to determine the cost for that timing, and then uses the cost as the "goodness" (or "fitness" in the language of genetic algorithms) of that timing. Using appropriately selected crossover and mutation operators, Jane then generates the next set of timings. The user can select the size of the sets to be used and the number of iterations (or "generations" in the language of genetic algorithms). Jane reports the best solutions discovered by the end of the last iteration.

Experimental results, reported later in this paper, demonstrate that Jane finds very good, and often optimal, solutions. While Jane is slower than Tarzan, it is fast enough to be used for large problems (e.g. optimal solutions for trees with 35-45 tips have been found in under one hour on a commodity personal computer). Moreover, the dynamic programming and genetic algorithm technique employed by Jane allow for a rich set of features that are not found in existing software packages for this problem. Among these unique features are:

• Ranges of time zones can be specified for divergence events in *both *the host and parasite trees.

• Upper bounds can be placed on the host switch distance, defined as the number of nodes passed from the takeoff to the landing site of a host switch. The significance of host switch distance, particularly for host-specific parasites, has been noted in several studies [10-12].

• Vertices in the host tree can be partitioned arbitrarily into regions and independent switch costs can be set between each pair of regions. As a special case, when every node in the host tree is in its own region this feature allows us to specify all possible host switch costs independently, for example based on host switch distance as suggested in [10].

• The graphical user interface allows the user to interactively modify solutions, with costs updated automatically, in order to explore the impact of perturbations on the computed solutions.

## Implementation

**Figure 3:**
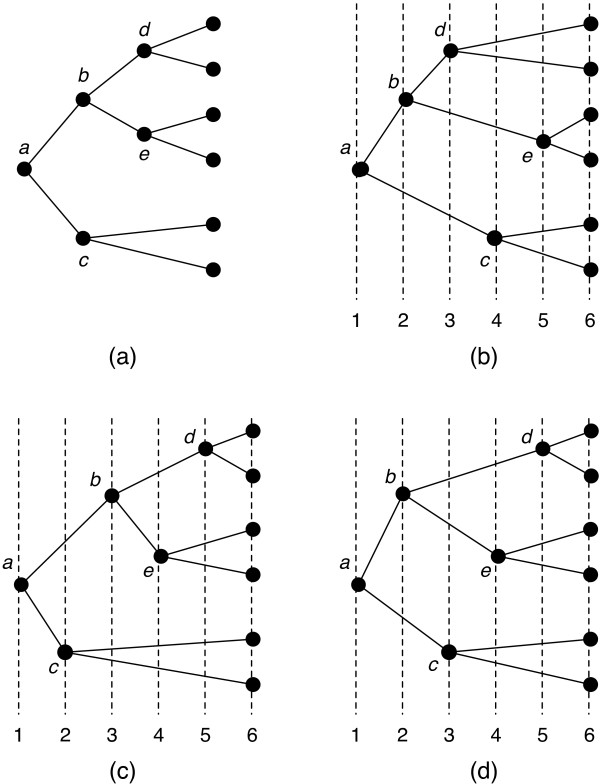
**(a) A host tree with three different timings shown in (b), (c), and (d)**. The numbers underneath each timing indicate the relative time of each vertex in that timing. The timings in (c) and (d) differ only in the relative times of nodes *b *and *c*, two nodes that occur at consecutive relative times but such that neither is the parent of the other. Thus, these two timings are said to be *neighbors*.

A *timing *of a host tree is an assignment of each internal vertex in the tree to a distinct relative time. The tips of the host tree are assumed to occur at the same relative time (current time). Figure 3(a) shows a host tree with the internal vertices labelled *a*, *b*, *c*, *d*, *e*. Three distinct timings are shown in Figure 3(b), (c), and 3(d).

Intuitively, a fixed timing for host tree events makes the problem computationally easier than the general problem because timing incompatibilities cannot arise in a fixed timing. For example, in the timing shown in Figure 3(b), a parasite associated with edge (*a*, *c*) can have a child on edge (*b*, *d*) via a host switch occurring between relative times 2 and 3. However, for the timing in Figure 3(c), this is not possible since node *c *occurs before node *b*.

Consider a timing and two vertices *x *and *y *occurring at consecutive relative times such that neither *x *nor *y *is the parent of the other. If the relative times of these two vertices are switched, the resulting timing is said to be a *neighbor *of the original timing. For example, in the timing in Figure 3(c), vertices *c *and *b *occur at consecutive relative times 2 and 3, respectively. Switching the relative times of these two vertices results in the timing in Figure 3(d).

Although the cophylogeny reconstruction problem is NP-complete, the problem can be solved optimally in polynomial time via dynamic programming, for any fixed timing in the host tree [1]. (The dynamic programming algorithm does not require that the events in the parasite tree have a fixed timing.) Unfortunately, the number of different timings grows exponentially with the number of tips, so the approach of examining all timings is not viable in general. However, a meta-heuristic approach can be used where we begin with a random timing for the host tree. We then solve the reconstruction problem optimally for this timing and compute the cost of this solution. Next, a neighbor timing is found and the problem is solved optimally for this timing. The policy for selecting neighbor timings and choosing which ones to keep and which to discard is dictated by the specific meta-heuristic.

For example, consider this approach with the simple *gradient descent *meta-heuristic. This heuristic begins by choosing an initial timing, *τ*. The dynamic programming algorithm is used to find the cost for each neighbor of *τ *and the neighbor that results in a least cost solution is chosen as the new timing *τ*. The process is repeated until a local minima is reached.

We experimented with gradient descent, simulated annealing, and genetic algorithms and found that a genetic algorithm approach consistently outperformed the others. In the genetic algorithm approach, we begin with an initial set of random timings for the host tree where the set has some given size *S*. For each timing, we solve the reconstruction problem optimally via dynamic programming to compute the cost for that timing. Next, two timings are chosen from the set at random, with repetition allowed, with probability weighted exponentially with fitness. Let *τ*_1 _and *τ*_2 _denote a specific chosen pair of timings. A crossover operator takes the two timings *τ*_1 _and *τ*_2 _and constructs a new timing, *τ*_new_, with elements from each of the two input timings. This process is performed until a new set of size *S *is constructed. The new set now replaces the previous set and the process is repeated until some stop condition is met. In our case the stop condition is a user-specified number of iterations.

We implemented and evaluated a number of different crossover operators. The most effective operator in our experiments, and thus the one implemented in Jane, is described next using an illustrative example.

**Figure 4:**
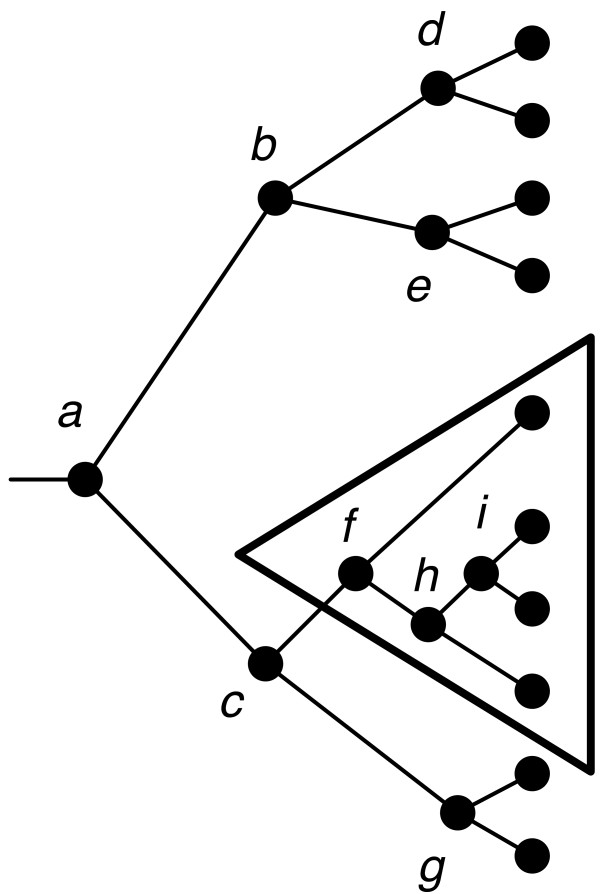
**The host tree with a randomly selected subtree**.

Two timings, *τ*_1 _and *τ*_2 _are selected at random from the current set as described above. A subtree, *T*, of the host tree is selected at random as shown in the example in Figure 4.

**Figure 5:**
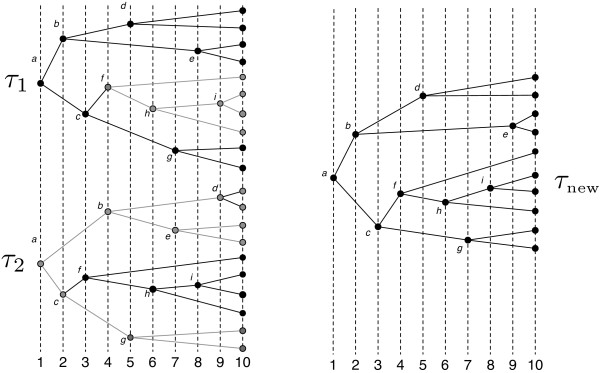
**At left, two selected timings, *τ*_1 _and *τ*_2_**. The subtree *T *is removed from *τ*_1 _while only *T *is kept in *τ*_2_, as indicated by grayed edges and vertices. The new timing *τ*_new _is shown on the right.

The selected subtree *T *is removed from one of the timings, *τ*_1_, as shown in the upper left of Figure 5, while only the subtree *T *is kept in the other timing, *τ*_2_, as shown in the lower left of Figure 5.

Now, we construct a new timing *τ*_new _by selecting relative times for each of its internal nodes. This is performed by constructing three lists, one for the nodes not in *T *ordered by their times in *τ*_1_, one for the nodes in *T *ordered by their times in *τ*_2_, and one initially empty list for the new timing *τ*_new _as shown in Table 1.

**Table 1:** Initial listing of nodes.

***τ*_1_**		***τ*_2_**		***τ*_new_**	
**node**	**time**	**node**	**time**	**node**	**time**
		
*a*	1	*f*	3		1
*b*	2	*h*	6		2
*c*	3	*i*	8		3
*d*	5				4
*g*	7				5
*e*	8				6
					7
					8
					9

The first time in *τ*_new _which needs a node assigned is time 1. To decide which node to select for this time, we examine the first nodes in the lists for *τ*_1 _and *τ*_2_. The candidates are *a *and *f*, but since node *f *'s parent, *c*, has not been assigned a time yet, we cannot consider it. Node *a *is therefore assigned to time 1 by default. This process is repeated and nodes *b *and *c *are placed in times 2 and 3. Table 2 shows the table at this point.

**Table 2:** Assignments from *τ*_1 _made such that the first node in *τ*_2 _can be assigned.

***τ*_1_**		***τ*_2_**		***τ*_new_**	
**node**	**time**	**node**	**time**	**node**	**time**
		
	1	*f*	3	*a*	1
	2	*h*	6	*b*	2
	3	*i*	8	*c*	3
*d*	5				4
*g*	7				5
*e*	8				6
					7
					8
					9

Now, *d *and *f *are candidates for time 4. In general, the algorithm chooses the candidate whose time is closest to the time under consideration. Since *d *is at time 5 in *τ*_1 _and *f *is at time 3 in *τ*_2_, they are equally close to time 4 and thus one is chosen at random. For this example, *f *is chosen for time 4.

Next, *d *and *h *are considered for time 5 in *τ*_new_. Since *d *has time 5 and *h *has time 6, *d *is chosen. Continuing in this fashion, the resulting timing for *τ*_new _is constructed and shown in Table 3 with the timing in the tree shown on the right side of Figure 5.

**Table 3:** Final assignment for all nodes in the new individual.

***τ*_1_**		***τ*_2_**		***τ*_new_**	
**node**	**time**	**node**	**time**	**node**	**time**
		
	1		3	*a*	1
	2		6	*b*	2
	3		8	*c*	3
	5			*f*	4
	7			*d*	5
	8			*h*	6
				*g*	7
				*i*	8
				*e*	9

This crossover operator was empirically found to preserve beneficial host switches and maintain a higher diversity of different solutions than other tested crossover operators. In order to introduce additional variation into the sets, some fraction of timings are selected for random mutation, where a mutation involves swapping the order of two nodes that occur at consecutive times and do not have a parent-child relationship. There are several parameters in this genetic algorithm including those in the probability function for selecting timings for crossover and the mutation rate.

## Results

Jane is implemented in Java and comes in a platform-independent jar file. The implementation is multi-threaded to take advantage of multi-core systems. The dynamic programming step used to evaluate a timing in the genetic algorithm is the primary contributor to Jane's running time. Since the genetic algorithm maintains a set of timings to be evaluated, multi-threading allows for near-linear speedup on multi-core systems.

**Figure 6:**
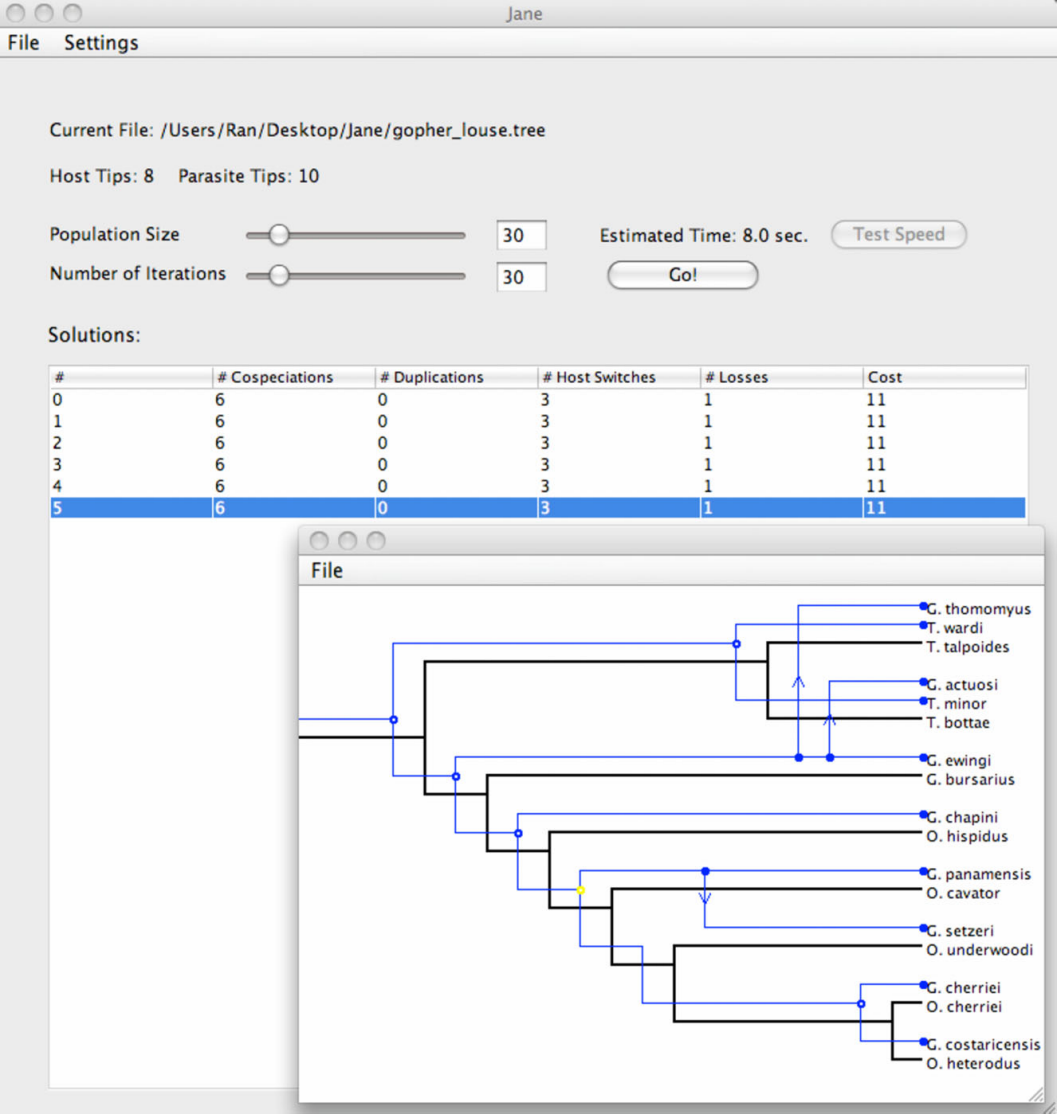
**The Jane graphical user interface with a selected solution shown in the inset window**.

Jane can be run interactively through a graphical user interface shown in Figure 6 or directly from the command line. The latter option is convenient for running large numbers of tests under the control of a script.

Jane has a number of user-definable parameters including event costs, host switch distance bound, and the number of iterations and the set size in the genetic solver. These parameters are all exposed to the user in the graphical user interface. Guidance in choosing the number of sets and set size, based on systematic experiments, is available on the Jane website [13].

Other parameters that are less likely to be of interest to the user include those for the rate at which timings are mutated in the genetic algorithm, among others. These parameters are not exposed in the graphical user interface but can be set in the command-line version of Jane. Values of these parameters were systematically evaluated and the best values found are used as defaults.

Jane can import its files in either Tarzan or a Nexus-based format. A file must specify the host and parasite trees and the tip associations. Optionally, the file can specify time zones or time zone ranges as well as regions (groups of nodes in the host tree) and the host switch costs between each pair of regions. Jane reports both the best solution found and a set of distinct timings that admit this solution. The user can select such a timing, see a graphical representation of the solution, and modify the solution by clicking on a parasite association on the host tree and moving it elsewhere on the host. When the parasite node is selected, Jane displays alternate association sites using three colors: yellow indicates that there is no increase in cost in moving the solution to this location, red indicates an increase in cost, and green indicates a decrease in cost. (Since only reports the best solutions found, green choices will not arise initially, but may arise after the user has first made some choices that increase the cost.)

### Experimental Results

We have examined the results found by Tarzan and Jane on six problem instances from the literature. The host and parasite trees in these problems ranged from 8 to 44 tips. In some problem instances, the host and parasite trees had different number of tips. For example, in some cases multiple parasite tips mapped to the same host tip while in other cases some host tips had no associated parasite tips. TreeMap was not used in these experiments because the exponential time and space that it requires precluded its use for most of the problem instances.

• Problem 1 is for pocket gophers and their chewing lice parasites [5,14]. The host tree has 8 tips and the parasite tree has 10 tips.

• Problem 2 is for seabird hosts (albatrosses, petrels, and penguins) and their ischnocrean lice [15]. The host tree has 11 tips and the parasite tree 14 tips, with some hosts tips associated with several lice.

• Problem 3 is for *Ficus *hosts and their *Ceratosolen *pollinators [16,17]. Each of these two trees has 16 tips.

• Problem 4 is for caryophyllaceous hosts and anther smut fungi parasites [18]. The host tree has 20 tips and the parasite tree has 24 tips.

• Problem 5 is for finch hosts (family Estrildae) and their African brood parasites *(Vidua) *[7,19]. The host tree has 33 tips and the parasite tree has 21 tips, with some host tips having no associated parasites.

• Problem 6 is for host plants (Leguminosae) and phytophagous insects (Psylloidae) on the Canary Islands [7,20]. The host tree has 36 tips and the parasite tree has 44 tips.

The event costs used here were 0 for cospeciation, 1 for duplication, 1 for host switching, and 2 for loss. (These costs are with respect to Charleston's cost scheme [5]. Tarzan uses a slightly different scheme and the appropriate conversion [7] was performed for correct comparisons with Tarzan.)

Jane permits the user to set the number of iterations (or "generations" in the language of genetic algorithms), *G*, and the size, *S*, for each set (or "population" in the language of genetic algorithms). The number of invocations of the dynamic programming algorithm is equal to the product of *G *and *S*. Since the dynamic programming step dominates the running time, it is desirable to keep this product as low as possible. We have performed extensive experimental studies to determine good choices for the values of these parameters and the results of these studies are summarized on the Jane website [13]. In brief, we have found that for the small to medium-sized trees in our examples, a good choice is to have *G *≈ 2*S *and use approximately 1000 runs of the dynamic programming solver. Solving for *G *and *S *under these assumptions, we chose *G *= 45 sets of size *S *= 23.

Jane incorporates randomness in several places: the selection of the set in the initial iteration, the random choice of timings and subtrees used in the crossover step, and random mutations of timings. Therefore, the best solutions found by Jane can potentially vary from run to run. To test the sensitivity to this randomness in the algorithm, we ran Jane 30 times for each problem (each run comprising 45 sets of set size 23).

The results of these computational experiments are summarized in Table 4. For Jane, the columns "Min", "Max", and "Mean" represent the minimum, maximum, and mean costs of the best solutions found over the 30 independent runs. Tarzan is entirely deterministic so the "Min" column there represents the best solution found by Tarzan. The results show that for the smaller trees in Problems 1 through 4, the randomness inherent in the genetic algorithm had no impact on the best solution found by Jane. However, for the larger trees in Problems 5 and 6, the randomness in the algorithm contributed to modest variability in the best solutions.

**Table 4:** Summary of experiments on six host-parasite problem instances.

		Tarzan		Jane			
Problem	Tips	Min	Time	Min	Max	Mean	Mean Time
1	18	11	< 1 sec	11	11	11.0	11.4 sec

2	25	20	< 1 sec	20	20	20.0	40.6 sec

3	32	20	< 1 sec	20	20	20.0	44.0 sec

4	44	50^+^	< 1 sec	51	51	51.0	743.9 sec

5	54	44	< 1 sec	44	47	44.1	2166.8 sec

6	80	98*	< 1 sec	99	105	101.13	4473.6 sec

Key: The second column indicates the sum of the number of tips in the host and parasite trees. The columns labeled "Min" indicate the best solutions found. Since Jane uses randomness, the columns "Max" and "Mean" indicate the worst and average optimal solutions found over 30 independent runs. The Tarzan solution marked with + used a type of host switch not permitted in Jane and the solution marked with an asterisk was incorrect due to strong timing incompatibilities. All experiments were performed on a commodity iMac Intel Core 2 Duo computer with clock speed of 2.66 GHz and 4 GB memory.

It should be noted that when Tarzan succeeds in finding a valid result, these results are, in theory, optimal. Thus, both Jane and Tarzan found optimal solutions in every case, with the exception of Problems 4 and 6. In the case of Problem 4, Tarzan reported solutions that used a host switching event that is not permitted in either Jane or TreeMap whereas in Problem 6 Tarzan reported a solution that was incorrect due to strong timing incompatibilities [9]. (Tarzan permits a parasite *p *to switch from host edge *e *to host edge *e' *at any time. TreeMap and Jane require that host switches occur contemporaneously with a duplication event.)

In addition, we examined several instances of the cophylogeny problems in the literature where solutions were reported using tools such as TreeMap and TreeFitter. We then analyzed those instances using Jane (with the same number of iterations and set size as in the experiments above) to see how the solutions compared. For example, we considered the results described by Hughes *et al*. [21] on the cophylogeny problem for maximum parsimony phylogenetic trees of pelecaniform birds and *Pectinopygus *lice. In that study, TreeMap and TreeFitter were used to find least cost solutions for these trees with 18 tips each. Using default cost settings, the optimal solutions found by both TreeMap and TreeFitter incurred 11 cospeciations, 0 duplications, 3 loss events, and 6 host switches. In contrast, using TreeMap's default cost settings, Jane found 19 optimal solutions all using 12 cospeciations, 0 duplications, 5 loss events, and 5 host switches. Under the TreeMap default cost settings, TreeMap's solution had cost 21 while Jane's solutions had cost 20. Using the default cost settings from TreeFitter, Jane found solutions with 11 cospeciations, 0 duplications, 2 loss events, and 6 host switches.

As a second example, we examined the case of petrel lice on the genus *Halipeurus *studied by Page *et al*. [22]. In this study, TreeMap was used to reconcile a parasite tree with 14 tips and a host tree with 13 tips (two parasites were associated with one host). Using its default cost settings, TreeMap reported a solution with 6 cospeciations, 4 duplications, 15 losses, and 2 host switches for a total cost of 25. All of Jane's solutions under these cost settings used 6 cospeciations, 1 duplication, 3 losses, and 6 host switches for a total cost of 23. While in theory TreeMap can find optimal solutions, Page *et al*. had to constrain the number of host switches to 3 in order to solve the problem in a reasonable amount of time and memory. For this reason, TreeMap did not discover the lower cost solutions found by Jane. While the running time of TreeMap was not reported for these experiments, Jane found its solutions in approximately two minutes. In general, Jane runs significantly faster than TreeMap and somewhat slower than Tarzan. The dominant component in Jane's running time is the dynamic programming (DP) solver which runs in time *O*(*n*^7^) where *n *is the total number of tips in the host and parasite trees. For example, for randomly generated host-parasite instances with 20 tips for each tree, the average running time of the DP was under 0.25 seconds and for 50 tips, the average running time of the DP was under 11 seconds on a 2.66 GHz Core 2 Duo iMac. For problems of this size, we found experimentally that the genetic algorithm requires approximately 1000 invocations of the DP to find optimal or near-optimal solutions. These results demonstrate that Jane is able to find very good, and often optimal, solutions within reasonable computation time.

## Conclusions

We have described the new Jane tool for the cophylogeny reconstruction problem. In contrast to TreeMap, Jane can solve much larger problem instances. In contrast to Tarzan, Jane always finds correct solutions. Jane also offers some features that are not found in existing software tools. For example, Jane allows the user to specify the maximum permitted host switch distance, where host switch distance is defined as the length of the path from the takeoff to the landing site of the switch in the host tree [10-12]. Additionally, the user may set different host switch costs for different regions of the host tree and set ranges of times in both the host and parasite trees. Jane offers a new graphical user interface that allows the user to explore solutions by interactively modifying them and seeing the impact on the solution cost. Finally, Jane supports an alternate command-line interface that allows for convenient implementation of large experiments under the control of scripting programs.

Existing software tools for the cophylogeny reconstruction problem use different algorithmic techniques and thus potentially produce different solutions. The practitioner may, therefore, find it valuable to use multiple tools to obtain a larger diversity of different results [21]. Moreover, each tool has some unique and important features.

## Availability and Requirements

Jane is implemented in Java with the Swing toolkit and runs on any machine with Java 1.5 or higher. The source code and documentation are freely available from the Jane website [13] and are distributed under the FreeBSD licensing agreement.

## Competing interests

The authors declare that they have no competing interests.

## Authors' contributions

CC implemented and analyzed various meta-heuristics and devised the crossover operator used in Jane. He was also instrumental in the design, implementation, testing of Jane. DF was responsible for large portions of the design, implementation, and testing of Jane as well as its documentation. YO was responsible for experiments and performance analyses. RLH developed the dynamic programming algorithm, the meta-heuristic approach for the cophylogeny reconstruction problem, and contributed to the functional specification of the Jane tool. All authors have read and approved this paper.
